# Inhibition of Renin Angiotensin Axis May Be Associated with Reduced Risk of Developing Venous Thromboembolism in Patients with Atherosclerotic Disease

**DOI:** 10.1371/journal.pone.0087813

**Published:** 2014-01-31

**Authors:** Young Kwang Chae, Danai Khemasuwan, Anastasios Dimou, Stefan Neagu, Lakshmi Chebrolu, Shikha Gupta, Alejandra Carpio, Jongoh Kim, Jeong Hyun Yun, Athanasios Smyrlis, Alan Friedman, William Tester

**Affiliations:** 1 Department of Cancer Medicine, University of Texas MD Anderson Cancer Center, Houston, Texas, United States of America; 2 Department of Medicine, Cleveland Clinic, Ohio, United States of America; 3 Department of Medicine, Albert Einstein Medical Center, Philadelphia, Pennsylvania, United States of America; College of Pharmacy, University of Florida, United States of America

## Abstract

**Background:**

Arterial and venous thrombosis may share common pathophysiology involving the activation of platelets and inflammatory mediators. A growing body of evidence suggests prothrombotic effect of renin angiotensin system (RAS) including vascular inflammation and platelet activation. We hypothesized that the use of angiotensin converting enzyme inhibitors (ACEIs) or angiotensin receptor blockers (ARBs) plays a role in protecting against venous thromboembolism (VTE) in patients atherosclerosis.

**Methods:**

We conducted a retrospective study, reviewing 1,100 consecutive patients admitted to a teaching hospital with a diagnosis of either myocardial infarction or ischemic stroke from 2005 to 2010. Patients who had been treated with anticoagulation therapy before or after the first visit were excluded. The occurrence of VTE during the follow up period, risk factors for VTE on admission, and the use of ACEIs or ARBs during the follow up period were recorded.

**Results:**

The mean age of the entire study population was 68.1 years. 52.0% of the patients were female and 76.5% were African American. 67.3% were on RAS inhibitorsThe overall incidence of VTE was 9.7% (n = 107). Among the RAS inhibitor users, the incidence of VTE events was 9.0% (54/603) for the ACEI only users, 7.1% (8/113) for the ARB only users, and 0% (0/24) for the patients taking combination of ACEI and ARB. Among patients on RAS inhibitors, 8.4% (62/740) developed a VTE, compared with 12.5% (45/360) in the nonuser group [HR (hazard ratio), 0.58; 95% CI (confidence interval), 0.39–0.84; P<0.01]. Even after controlling for factors related to VTE (smoking, history of cancer, and immobilization, hormone use) and diabetes, the use of RAS inhibitors was still associated with a significantly lower risk of developing VTE (AHR, 0.59; 95% CI, 0.40–0.88; P = 0.01).

**Conclusions:**

The use of RAS inhibitors appears to be associated with a reduction in the risk of VTE.

## Introduction

Venous thromboembolism (VTE) is a serious condition affecting approximately 2 persons per 1000 each year [1], [2]. Although traditional risk factors as well as hereditary disorders have been identified, one third of cases are classified as idiopathic in etiology and questions regarding its pathophysiology still remain to be answered. Pathophysiology of venous thromboembolism (VTE) was thought to be different from thrombotic atherosclerosis. However, recent evidence indicates a possible common mechanism between VTE and atherosclerotic disease. For example, inflammatory cytokines play an important role in both venous and arterial thrombosis. Internleukin-6 (IL-6), IL-8 and tumor necrosis factor alpha (TNF-α) released by the inflammatory cells present in the atherosclerotic plaques [3], [4] are also found to be elevated in patients with venous thrombosis [5], [6]. In addition, platelet activation and adhesion plays a role not only in arterial thrombosis but also in venous thrombosis. Male smokers were found to have an increased platelet adhesion which translated into higher incidence of pulmonary embolism (PE) [7].

Patients with idiopathic VTE were shown to have a higher prevalence of asymptomatic carotid plaques [8] and coronary artery calcification [9]. Interestingly, they had an increased risk of subsequent cardiovascular events [10]. Likewise, patients with history of myocardial infarction or stroke had significantly increased risk for VTE within 3 months after the diagnosis [11]. In addition, a significant portion of patients with VTE had major cardiovascular risk factors such as metabolic syndrome, abdominal obesity, and abnormal lipid profiles [12]. However, two prospective studies have demonstrated no association between the risk of VTE and the presence of risk factors for thrombotic atherosclerosis [13], [14].

A growing body of evidence suggests prothrombotic effect of renin angiotensin system (RAS) [15], [16] Evidence for the protective role of some RAS inhibitors against atherothrombotic cardiovascular disease is already well established [16]. In fact, RAS inhibitors demonstrated a risk reduction of VTE as well as arterial thrombosis in animal studies [17], [18]. Given the possible common pathophysiology behind VTE and thrombotic atherosclerosis, we hypothesized that the use of ACEIs or ARBs, therefore, plays a role in protecting against VTE in patients with history of atherosclerosis. To our knowledge, whether ACEIs or ARBs actually prevents VTE has not been studied in a clinical setting.

## Methods

### Ethics statement

The study protocol was reviewed by the Albert Einstein Healthcare Network Institutional Review Board. Given the retrospective nature of the study, it was not possible to obtain written consents for participation in the study. The need for written consents was waived by the Institutional Review Board of the hospital on the basis of minimal risk to human subjects. Information was revealed to human subjects where appropriate after participation in the study.

### Patients and data collection

We conducted a retrospective cohort study in patients with established diagnosis of atherosclerosis defined in our study by ischemic stroke or myocardial infarction (MI). The start day of the cohort is the first day of admission for ischemic stroke or MI (the first visit). The diagnosis of transient ischemic attack or ischemic stroke was made using established criteria including a history of sudden onset, focal or global neurological deficits and confirmed by computerized tomography or magnetic resonance imaging scans. MI was determined by a typical rise and/or gradual fall of biochemical markers (troponin I, CK-MB) of myocardial necrosis, with at least one of the following: ischemic symptoms or ECG changes indicative of ischemia (ST segment elevation or depression) or imaging evidence of new loss of viable myocardium or a new regional wall motion abnormality [19]. Patient data with the above diagnoses admitted to Albert Einstein Medical Center (AEMC) between 2005 and 2010 were collected. All data were retrieved from electronic medical record system. The occurrence of VTE during the follow up period, possible risk factors for VTE on admission as well as demographic variables, and the use of RAS inhibitors during the follow up period were recorded. A data extraction sheet was designed to extract this information. To minimize subjective judgment and selection bias, investigators were blinded to outcomes. Regarding medication use, patients who never used RAS inhibitors or had used them for less than 2 months were assigned to the nonuser group. Patients who have used either ACEi or ARBs or both classes of drugs for at least 2 months were classified in the user group. All patients included into analysis had at least 2 hospital visits including the first visit. Patients were followed up to maximum of 6 years. Patients who had been treated with anticoagulation therapy before or after the first visit at AEMC were excluded. Patients with any previous history of VTE were excluded as well. The outcome of our study is the occurrence of VTE, which consist of DVT and/or PE. Doppler ultrasound using established major criteria of venous compressibility with transducer was used to diagnose DVT of femoral and popliteal veins. Computerized tomographic pulmonary angiography with the presence of PE or high probability on ventilation-perfusion scan was used to diagnose pulmonary embolism. The study protocol was reviewed and approved by the Institutional Review Board of Albert Einstein Medical Center.

### Statistical analysis

Data were collected into a data spread sheet and transferred to STATA (version 9, College Station, TX). The Kaplan-Meier method was used to estimate time to VTE for the RAS inhibitor user group and the nonuser group. The log rank test was used to compare differences in time to VTE between the RAS inhibitor user group and the nonuser group. Baseline characteristics at the first visit were compared within each group using either an analysis of variance for continuous variables or the chi-square test for categorical data. All P values were 2-sided with a level of 0.05 for statistical significance. Multivariable analyses were performed using Cox proportional hazards regression. Independent variables included diabetes mellitus, obesity (BMI more than 30), smoking, history of cancer, history of metastatic cancer, immobilization, and use of estrogen hormone or its derivatives.

## Results

1,795 patients with the new diagnosis of either MI or ischemic stroke were identified from 2005 to 2010. Among this group, patients that were treated with anticoagulation therapy (therapeutic dosage of intravenous heparin or oral warfarin) before their first admission or patents that received anticoagulation therapy after the diagnosis of cardiovascular diseases were excluded (n = 208). In addition, patients who had single hospital visit were excluded (n = 487). This resulted in a total of 1,100 patients for the final analysis.

The mean age of the entire study population was 68.1 years46.5% of the patients were female and 76.1% were African American. Among 1100 patients, 740 (67.3%) were using RAS inhibitors: either ACEIs only (n = 603, 54.8%) or ARBs only (n = 113, 10.3%) or both (n = 24, 2.2%). Demographic and Clinical characteristics are compared between the ACEI/ARB user and nonuser groups (Table 1). As expected, ACEI/ARB users had higher prevalence of hypertension and diabetes. They had less smokers and they were younger (Table 1).

**Table 1:** Comparison of clinical variables between ACEI/ARB users and nonusers.

Variables	Total (n = 1,100)	ACEI/ARB user (n = 740)	ACEI/ARB nonuser (n = 360)	p value
Age, mean (SD), y		66.4 (14.1)	69.0 (15.5)	<0.01
Gender				
Male	578 (52.5%)	399 (53.9%)	179 (49.7%)	0.19
Female	522 (47.5%)	341 (46.1%)	181 (50.3%)	
Race				
African-American	838 (76.2%)	574 (77.6%)	264 (73.3%)	
Caucasian	165 (15%)	102 (13.8%)	63 (17.5%)	0.11
Hispanic	56 (5.1%)	41 (5.5%)	15 (4.2%)	
Asian	41 (3.7%)	23 (3.1%)	18 (5%)	
Smoking history				
smoker	750 (68.2%)	490 (66.2%)	260 (72.2%)	0.03
nonsmoker	350 (31.8%)	250 (33.8%)	100 (27.8%)	
Hypertension				
Yes	1015 (92.3%)	701 (94.7%)	314 (87.2%)	<0.01
No	85 (7.7%)	39 (5.3%)	46 (12.8%)	
Diabetes				
Yes	572 (52%)	418 (56.5%)	154 (42.8%)	<0.01
No	528 (48%)	322 (43.5%)	206 (57.2%)	
Cancer				
Yes	184 (16.7%)	114 (15.4%)	70 (19.4%)	0.06
No	916 (83.3%)	626 (84.6%)	290 (80.6%)	
Metastasis				
Yes	45 (4.1%)	22 (3.0%)	23 (6.4%)	0.01
No	1055 (95.9%)	718 (97.0%)	337 (93.6%)	
Immobilization				
Yes	249 (22.6%)	161 (21.8%)	88 (24.4%)	0.32
No	851 (77.4%)	579 (78.2%)	272 (75.6%)	
Obesity				
Yes	266 (24.2%)	190 (25.7%)	76 (21.1%)	0.1
No	834 (75.8%)	550 (74.3%)	284 (78.9%)	
Hormone Use				
Yes	10 (0.9%)	7 (1.0%)	3 (0.9%)	0.85
No	1090 (99.1%)	733 (99%)	357 (99.1%)	
PVD				
Yes	97 (8.8%)	58 (7.8%)	39 (10.8%)	0.1
No	1003 (91.2%)	682 (92.2%)	321 (89.2%)	
VTE				
Yes	107 (9.7%)	62 (8.4%)	45 (12.5%)	0.03
No	993 (90.3%)	678 (91.6%)	315 (87.5)	

ACEI; angiotensin converting enzyme inhibitor, ARB; angiontensin receptor blocker, SD; standard deviation, VTE; venous thromboembolism.

**Figure 1:**
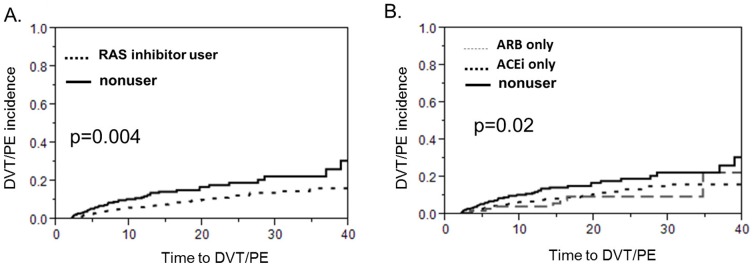
Kaplan-Meier venous thromboembolism (VTE) free survival curve between (angiotensin converting enzyme) ACE inhibitor or angiotensin receptor blocker (ARB) users and nonusers. As shown in A, patients with either stroke or MI who use ACE inhibitors or ARBs have a longer VTE free survival compared to the patients who use none of these drugs. On the other hand there is no difference between the ACE inhibitor only users and the ARB only users with regards to VTE free survival, as the corresponding curves in B overlap.

**Figure 2:**
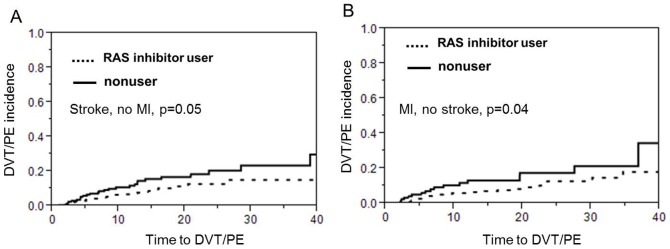
The protective effect of ACE inhibitors or ARBs is sustained in the subgroup of patients who experienced a stroke but did not have a myocardial infarction (MI) as shown in A and in the subgroup of patients who had an MI but not a stroke as shown in B.

**Figure 3:**
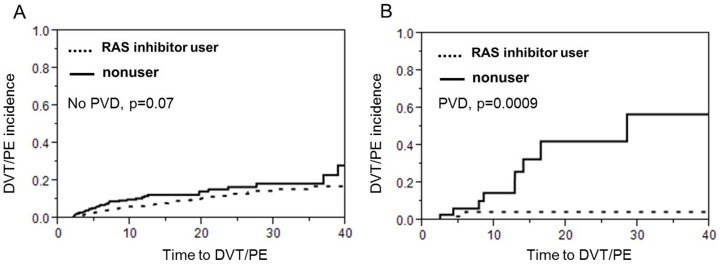
Among patients with either MI or stroke who had a concomitant diagnosis of peripheral vascular disease (PVD) there was a prominent protective effect of ACE inhibitors or ARBs against VTE as shown in A but this was not true in the patients with either stroke or MI who did not have concomitant diagnosis of PVD as shown in B.

The overall incidence of VTE was 9.7% (n = 107). 80.3% (n = 86) had DVT and 30.8% (n = 33) had PE. 11.2% (n = 12) had both. Among patients on RAS inhibitors, 8.4% developed a VTE, compared with 12.5% in the nonuser group [HR (hazard ratio), 0.58; 95% CI (confidence interval), 0.39–0.84; P<0.01]. Kaplan-Meier VTE free survival analysis displayed reduced risk of VTE for RAS inhibitor users compared to nonusers (Figure 1A, log rank test p = 0.004 ). Metastasis, obesity, and estrogen hormone use were positively associated with VTE (Table 2). After controlling for factors related to VTE (smoking, history of cancer, and immobilization, hormone use), age and diabetes, the use of RAS inhibitors was still associated with lower risk of developing VTE [AHR (adjusted hazard ratio), 0.60; 95% CI, 0.40–0.90 ; P = 0.014]. Interestingly, among patients using both ACEI and ARB, no one developed VTE (0/24), compared with 9.0% (54/603) in ACEI only users and 7.1% (8/113) in ARB only users (chi square test p = 0.06, data not shown). However, Kaplan-Meier VTE survival analysis displayed did not show any difference between the ACEi only group and the ARB only group. On the other hand the RAS inhibitor users had a longer VTE survival time compared to non-users in the subgroup of patients who were initially admitted for stroke only (p = 0.05, Figure 2A) and in the subgroup of patients who were admitted for MI only (p = 0.04, Figure 2B). Last but not least, the protective effect of ACEi/ARBs with regards to VTE was present in the patients who had a concomitant diagnosis of peripheral vascular disease (p = 0.0009, Figure 3B) but only trending in the subgroup of patients with no peripheral vascular disease (p = 0.07, Figure 3A).

**Table 2:** Association between VTE and clinical variables including RAS inhibitor use.

Variables	HR	95% CI	p value	AHR	95% CI	p value
**Age (continuous)**	1.01	0.99, 1.02	0.06	0.85	0.56, 6.25	0.31
**Diabetes**	1.00	0.68, 1.46	0.99	0.95	0.63, 1.42	0.80
**Hypertension**	2.92	0.93, 9.22	0.07	2.92	1.08, 12.02	0.03
**Smoking**	0.72	0.47, 1.10	0.13	0.82	0.50, 1.30	0.40
**Cancer**	1.40	0.88, 2.24	0.16	1.02	0.53, 1.78	0.94
**Metastasis**	2.34	1.20, 4.71	0.02	2.35	0.94, 5.59	0.06
**Immobilization**	2.01	1.35, 2.98	0.11	1.88	1.24, 2.79	0.002
**Obesity**	1.55	1.05, 2.29	0.03	1.64	1.08, 2.46	0.019
**Hormone**	3.25	1.32, 33.60	0.04	2.35	0.55, 6.71	0.21
**RAS inhibitor use**	0.58	0.39, 0.84	0.006	0.60	0.40, 0.90	0.014

hazard ratio, CI; confidence interva.

## Discussion

We have found that the use of RAS inhibitors may be related to a reduced risk of VTE in patients with atherosclerotic disease. The absolute risk reduction in VTE for RAS inhibitor users was 4.1% over the period of our study. With previous in vitro and in vivo findings [16] suggesting thrombotic effect of RAS activation, our results may indicate that the use of ACEIs or ARBs may prevent the occurrence of VTE in high risk patients such as patients with known history of MI or ischemic stroke as in our study population. The protective effect of RAS inhibitors with regards to VTE is more pronounced in patients with a concomitant diagnosis of peripheral vascular disease. Interestingly, peripheral vascular disease correlates with a higher burden of atherosclerosis [20]. To our knowledge, this is the first clinical study that assessed the antithrombotic property of RAS inhibitors with respect to VTE prevention.

There are multiple mechanisms to explain our results. In vitro and in vivo data renders compelling evidence for an interaction between the RAS and thrombosis [15]. First, numerous deleterious effects of angiotensin II have been reported. Those include vasoconstriction, sympathetic nervous activation, smooth muscle cell growth and proliferation, vascular inflammation, generation of reactive oxygen species (ROS) and endothelial dysfunction. These effects are mediated through the angiotensin II type 1 (AT1) receptor. Angiotensin II also opposes the effect of nitric oxide, stimulates the production of platelet adhesion factors and plasminogen activator inhibitor-1 (PAI-1). Besides elevation in blood pressure, these effects are clinically translated into higher risk of thrombosis [16]. It is reasonable to surmise that use of angiotensin coverting enzyme inhibitor (ACEI) that lowers that serum level of angiotensin II and the use of angiotensin II receptor type 1 blocker (ARB) will have possible antithrombotic effect. Second, RAS inhibitors have been shown to increase blood flow to both capillary and venous system [21]. Since venous stasis is an important risk factor for VTE, protective effect of RAS inhibition can be explained by the improvement of blood rheology.

Other medications have been suggested to have possible detrimental or protective effect regarding VTE. For example, oral contraceptives and hormone replacement therapy have been associated with having higher risk of VTE [22]. Our study also found that hormone use was positively associated with VTE occurrence (Table 2). It is notable that adjusting for hormone use did not make any difference in the relation between ACEI/ARB use and the risk of VTE. Statins have been shown to have anti-inflammatory and antithrombotic effect and has been linked with reduced risk of VTE [23]–[25]. Antiplatelet agents including aspirin have also shown to be efficacious in preventing VTE [26]. In our study, statins and antiplatelet agents have shown negative association with VTE risk consistent with previous studies (data not shown). However, there was no interaction between the ACEI/ARB use and statins or antiplatelet agents (statins: Wald test p = 0.10, LHR test p = 0.11; antiplatelet agents: Wald test p = 0.14, LHR test p = 0.14).

There are several innate limitations of our study. First, it is a hospital based retrospective study. There could be missed confounding factors not included in our study that may have resulted in a differential loss to follow up. More comorbid conditions in ACEI/ARB users such as congestive heart failure or diabetic nephropathy may have played a role in this regard. Second, there could be a selection bias. For example, diabetic patients, especially ones with diabetic nephropathy, are recommended to use ACEI/ARB rather than other antihypertensives. Microalbuminuria as well as mild to severe chronic kidney disease prevalent in diabetic nephropathy has been reported to be associated with higher risk of VTE [27], [28]. However, this would underestimate than overestimate the preventive effect of ACEI/ARBs. To the contrary, people with contraindications for use of ACEI/ARBs such as acute kidney injury may have different VTE risk profile. Loss to follow up for 487 out of 1795 patients with either a CVA or an MI who were originally screened and did not have more than one visit in total might introduce additional selection bias. Third, there could be ascertainment bias. We used medication reconciliation form to assess the duration of ACEI/ARB use. However, it may not accurately reflect patients' medication adherence. Fourth, we were not able to assess the dose related effect in our study partly due to the fact that many people had frequent dose changes over the follow up period time. Lastly, the results cannot be generalized to people without atherosclerosis. Given the relationship between VTE and atherosclerosis as well as the protective effect of ACEi/ARBs on atherosclerosis the negative association between ACEi/ARBs and VTE risk that we found in our study is subject to collider stratification bias. Since our study was performed among patients with known ischemic CVA or MI, larger trials are needed to explore the effect of ACEI/ARBs in general population.

In summary, the use of RAS inhibitors appears to be associated with a reduction in the risk of VTE. This corroborates the existing in vitro and in vivo studies suggesting the antithrombotic property of ACEI or ARBs. This possible antithrombotic effect of antagonizing RAS in relation to VTE risk warrants further prospective clinical investigation.
